# Effects of bradykinin on proliferation, apoptosis, and cycle of glomerular mesangial cells via the TGF-β1/Smad signaling pathway

**DOI:** 10.3906/biy-2007-58

**Published:** 2021-02-09

**Authors:** Ji DONG, Li DING, Liuwei WANG, Zijun YANG, Yulin WANG, Ying ZANG, Xuexia CAO, Lin TANG

**Affiliations:** 1 Department of Medicine, Henan Medical College, Zhengzhou, Henan Province China; 2 Henan Institute for Occupational Medicine, Zhengzhou, Henan Province China; 3 Department of Nephropathy, The First Affiliated Hospital of Zhengzhou University, Zhengzhou, Henan Province China

**Keywords:** Glomerular mesangial cell, bradykinin, TGF-β1, Smad, proliferation, apoptosis, cell cycle

## Abstract

We aimed to assess the effects of bradykinin (BK) on the proliferation, apoptosis, and cycle of glomerular mesangial cells via the transforming growth factor-β 1 (TGF-β1)/Smad signaling pathway. Rat glomerular mesangial cells, HBZY-1, were divided into normal group (untreated), model group (5 ng/L TGF-β1), BK group (5 ng/L TGF-β1 + 1 ng/L BK), and inhibitor group [5 ng/L TGF-β1 + 1 ng/L LY2109761 (TGF-β1-specific inhibitor)]. The cell proliferation, cycle, apoptosis, expression of type I collagen (Col-1), and protein expressions of Col-1, TGF-β1, and phosphorylated Smad2 (p-Smad2) were detected by EdU labeling, flow cytometry, acridine orange/ethidium bromide (AO/EB) dual staining, immunofluorescence assay, and Western blotting, respectively. Compared with the normal group, the cell proliferation rate (P = 0.02) and protein expression levels of Col-1 (P = 0.02), TGF-β1 (P = 0.01), p-Smad2 (P = 0.02), and p-Smad7 (P = 0.00) in the model group significantly increased, and apoptosis rate (P = 0.01) significantly decreased. Compared with the model group, the BK and inhibitor groups significantly decreased in proliferation rate (P = 0.01) and protein expression levels of Col-1 (P = 0.01), TGF-β1 (P = 0.01), and p-Smad2 (P = 0.00). Also, they were significantly elevated in apoptosis rate (P = 0.02) and p-Smad7 protein expression (P = 0.02). BK regulates the proliferation, apoptosis, and the cycle of glomerular mesangial cells by inhibiting the TGF-β1/Smad signaling pathway.

## 1. Introduction

Glomerular mesangial cells are primary effector cells for the progression of chronic progressive kidney diseases into renal fibrosis (Lee et al., 2018). Mesangial cells are activated by multiple factors-triggered inflammatory cytokines to cause hyperproliferation, which enlarges the kidney mesangial area and decreases the glomerular filtration rate, thus forming a pathological state of sclerosis (Nguyen et al., 2019). Therefore, suppressing the abnormal proliferation of glomerular mesangial cells is crucial for the treatment of chronic kidney disease and chronic renal insufficiency, as well as the prevention and treatment of renal fibrosis. Bradykinin (BK) is an inflammatory mediator. Liu et al. (2018) found an increase in BK expression on the surface of glomerular mesangial cells in rats with chronic kidney disease. BK obviously affects glomerulosclerosis, but the mechanism remains unclear (Qin et al., 2019). Transforming growth factor-β 1 (TGF-β1) is the primary mediator of glomerulosclerosis leading to renal interstitial fibrosis (Song et al., 2017). As a strong fibrogenic factor, TGF-β1 has increased expression in the case of chronic glomerulitis (Pereira et al., 2010). Besides, it transfers extracellular signals into the nucleus based on proteins of the Smad family to cooperate with the expressions of related genes, which directly promotes the synthesis of type I collagen (Col-1) and the progression of glomerular fibrosis. Sen et al. (2019) confirmed that suppressing the TGF-β1 signaling pathway remarkably mitigated renal fibrosis induced by chronic renal insufficiency. In addition, Catalán et al. (2019) verified that BK resisted the fibrosis of rat cardiomyocytes by evidently inhibiting TGF-β1 signal. However, the roles of BK and TGF-β1 in kidneys have not been reported until now. In this study, rat glomerular mesangial cells were cultured in vitro to investigate the antifibrotic mechanism of BK based on TGF-β1/Smad signals, aiming to provide theoretical support for clinical practice.

## 2. Materials and methods

### 2.1. Cells, reagents, and apparatus

Rat glomerular mesangial cell line HBZY-1 was provided by the Cell Bank of Chinese Academy of Sciences (China). Fetal bovine serum (FBS), trypsin, and RPMI-1640 medium were purchased from Gibco Laboratories (Gaithersburg, MD, USA). Rabbit antihuman Col-1, TGF-β1, phosphorylated Smad2 (p-Smad2), and p-Smad7 antibodies and horseradish peroxidase (HRP) were bought from Abcam Inc. (Cambridge, MA, USA). EdU kit, bicinchoninic acid (BCA) protein concentration detection kit, acridine orange/ethidium bromide (AO/EB) staining kit, and Western blotting kit were purchased from Sigma-Aldrich Corp. (St. Louis, MO, USA). TGF-β1-specific inhibitor LY2109761 was provided by Nanjing Jiancheng Bioengineering Institute (China). The main apparatus included a CO_2_ cell incubator (Beckman Coulter, Inc., Brea, CA, USA), MK3 microplate reader (Shimadzu Corporation, Kyoto, Japan), a flow cytometer (Shel Lab; Sheldon Manufacturing, Inc., Cornelius, OR, USA), LIOOS600T fluorescence microscope (Nikon Corp., Tokyo, Japan), and a high-speed refrigerated centrifuge (Beijing Liuyi Biotechnology Co., Ltd., Beijing, China).

### 2.2. Cell culture and experimental grouping

HBZY-1 cells were centrifuged after the addition of RPIM-1640 medium containing 20% FBS to remove supernatant. Afterwards, the cells were suspended using culture medium, inoculated in a culture bottle with 5% CO_2_ at 37 °C and digested with 0.25% trypsin when the cell density reached 85%, followed by subculture in complete medium. Then, the cells in the logarithmic growth phase were assigned into normal group (untreated), TGF-β1 stimulation group (model group, 5 ng/L TGF-β1), BK + TGF-β1 stimulation group (BK group, 5 ng/L TGF-β1 + 1 ng/L BK), and TGF-β1-specific inhibitor LY2109761 stimulation group (inhibitor group, 5 ng/L TGF-β1 + 1 ng/L LY2109761).

### 2.3. Detection of cell proliferation by EdU labeling

The cells were inoculated into a 6-well culture plate at the concentration of 5 × 10^5^/well. Different groups of cells were treated in the same way as indicated above, and the concentration of EdU working solution was adjusted to 20 μmol/L. Then, 1 mL of EdU working solution was added to the cell culture plate, mixed gently, and cultured in a 5% CO_2_ incubator at 37 °C for 2 h. After the supernatant was discarded, the cells were fixed with 4% formaldehyde for 30 min. After being washed by phosphate-buffered saline (PBS) twice (5 min each time), the cells were left still in PBS with 0.3% Triton X-100 for 20 min at constant temperature. Subsequently, click reaction solution was prepared according to the kit’s requirements, and 500 μL of the reaction solution was added into each well with a pipette for incubation at constant temperature for 20 min. Then, the cells were incubated in 1 mL of DAPI solution for 30 min in the dark, washed with PBS, and observed under the fluorescence microscope. Ultimately, images were obtained to analyze the cell proliferation rate.

### 2.4. Detection of cell cycle by flow cytometry

The cells were treated in the same way as indicated above. They were washed with precooled PBS, suspended with 300 μL of buffer, and inoculated in a 24-well plate at the concentration of 1 × 10^6^/mL. After being cultured in the 5% CO_2_ incubator at 37 °C for 24 h, 1 mL of PBS was utilized to prepare a single cell suspension which was then dripped into 5 mL of 75% ethanol at 4 °C and fixed at 4 °C overnight. Subsequently, centrifugation was conducted at 1,000 r/min for 2 min to separate the liquid. The lower layer of the suspension was obtained, washed with PBS 3 times, and prepared into a cell suspension, using 200 μL of Annexin V-FITC binding solution. Then, 20 μL of PI staining solution was added and shaken gently at 200 r/min for 2 min, followed by placement in ice bath in the dark for 45 min. Finally, cell cycle changes were detected by the flow cytometer.

### 2.5. Detection of cell apoptosis by AO/EB dual staining

The cells were seeded into the 6-well culture plate at the concentration of 5 × 10^5^/well, and treated in the same way as above. After being cultured in the 5% CO_2_ incubator at 37 °C for 24 h, the supernatant was discarded on the next day, and the cells were rinsed by 20 mL of PBS 3 times and prepared into a single cell suspension. Then, 60 μL of freshly prepared AO/EB fluorescent staining solution (equal volumes of 100 mg/L AO and 100 mg/L EB) and 2 mL of PBS were added. The resulting mixture was excited at 388 nm under the fluorescence microscope, and cell apoptosis was observed.

### 2.6. Detection of Col-1 expression by immunofluorescence assay

The cells were inoculated in a 6-well culture plate at the concentration of 5 × 10^5^/well and treated in the same way as above. After being cultured in the 5% CO_2_ incubator at 37 °C for 24 h, the cells were fixed with 10% paraformaldehyde and prepared into ultrathin frozen sections according to the instructions of the immunofluorescence kit. Under the fluorescence microscope, red fluorescence indicated an expression of Col-1 (Weng et al., 2019).

### 2.7. Detection of Col-1, TGF-β1, p-Smad2, and p-Smad7 protein expressions by Western blot

The cells in different groups were treated in the same way as above, seeded into the 24-well plate at the concentration of 1 × 10^4^/well, and cultured in the 5% CO_2_ incubator at 37 °C for 24 h. After the cells were collected, target proteins Col-1, TGF-β1, p-Smad2, and p-Smad7 were extracted, and their expressions were measured by BCA kit. Subsequently, 50 μg of proteins were subjected to sodium dodecyl sulfate-polyacrylamide gel electrophoresis, after which the protein samples were transferred onto a polyvinylidene fluoride membrane by wet transfer method. Then, the membrane was blocked with 10% skimmed milk for 3 h and incubated with primary antibodies (1:500 diluted) at 4 °C overnight. After being washed, the membrane was incubated with HRP-labeled secondary antibodies (1:5000 diluted) for 3 h, followed by 30 min of color development by using enhanced chemiluminescence reagent. After image exposure, development and fixation, the protein expression level was represented by using GAPDH as the internal reference.

### 2.8. Statistical analysis

SPSS 16.0 software was employed for data analysis, and GraphPad Prism 5.01 software was used for plotting. Comparisons among multiple groups were conducted by univariate analysis, and the t-test was applied for pairwise comparisons. P < 0.05 indicated a statistically significant difference.

## 3. Results

### 3.1. Cell proliferation capacities

**Figure 1:**
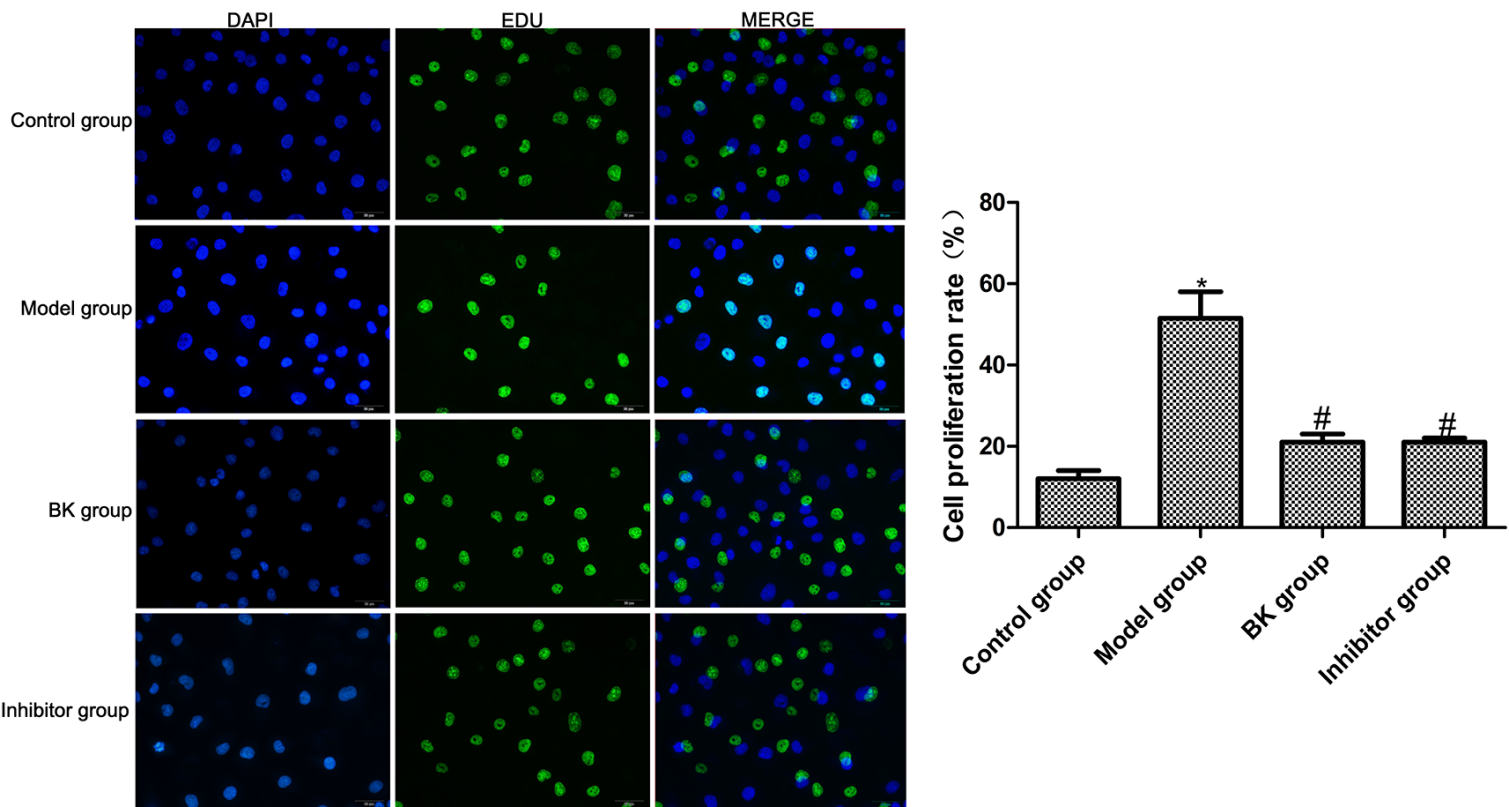
Cell proliferation capacities detected by EdU labeling ( x ± s , n = 5). Compared with normal group, *P < 0.05; compared with model group, #P < 0.05.

The EdU labeling assay showed that compared with the normal group, the proportion of EdU-positive HBZY-1 cells and cell proliferation rate in the model group increased significantly after TGF-β1 stimulation (P = 0.02). Compared with the model group, the proportion of EdU-positive cells and cell proliferation rate in BK group and inhibitor group decreased significantly (P = 0.01), but the latter two groups had similar results (P = 0.09) (Figure 1).

### 3.2. Cell cycle distribution

**Figure 2:**
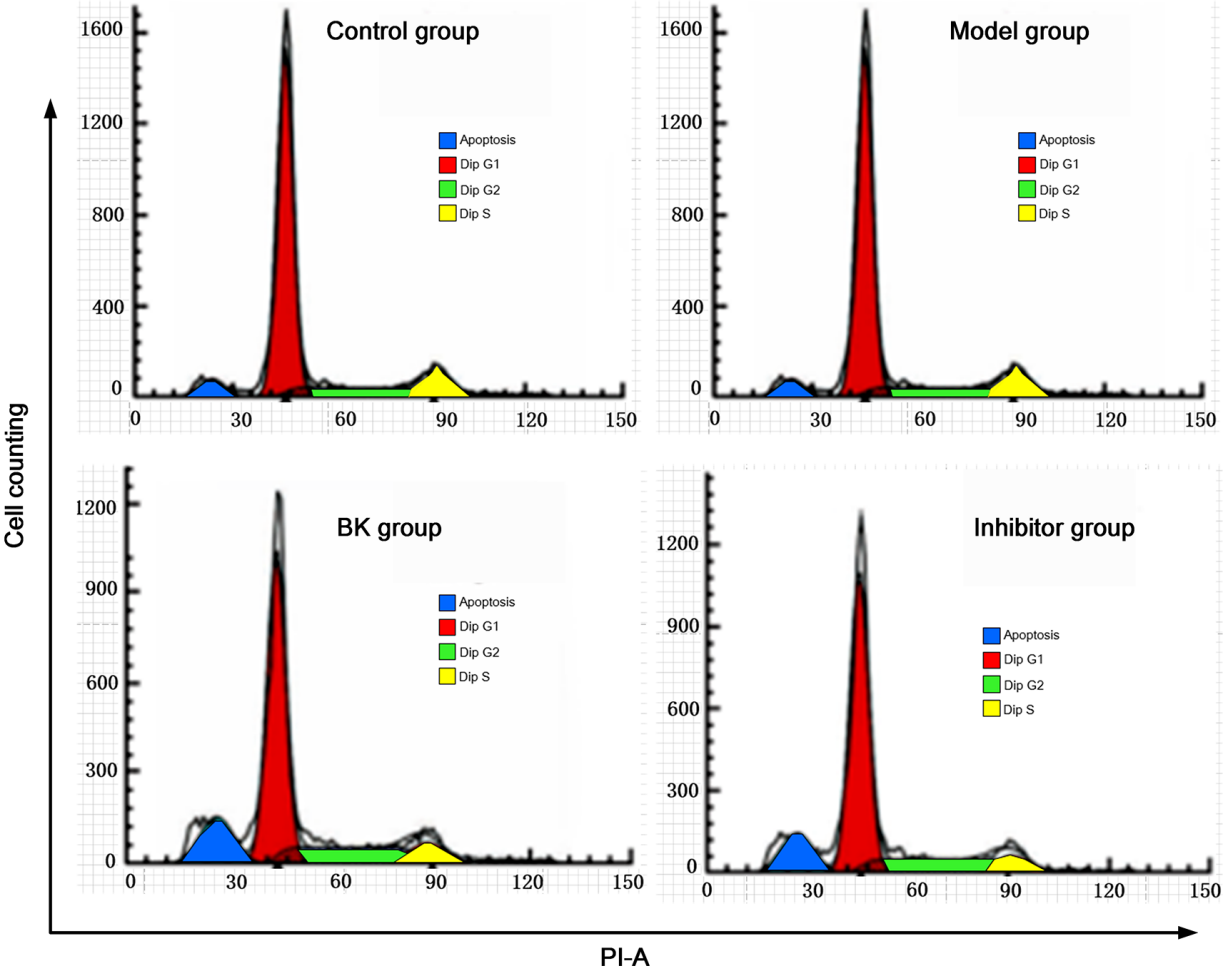
Cell cycle distribution detected by flow cytometry ( x ± s , n = 5).

Flow cytometry revealed that the cell cycle of the normal group hardly changed. Compared to the normal group, the percentage of cells in G1 phase decreased and that in G2/S phase increased significantly in the model group (P = 0.01), indicating increased protein synthesis and raised proportion of cells in the division stage. Compared with the model group, BK and inhibitor groups had significantly elevated the percentage of cells in G1 phase and reduced percentage in G2/S phase (P = 0.01), suggesting cell cycle arrest in G1 phase (Table and Figure 2).

**Table:** Table. Apoptotic rate and cell cycle distribution (, n = 5).

Group	G1 phase	G2/S phase
Normal	78.31 ± 0.22	17.48 ± 0.92
Model	55.08 ± 3.21*	42.61 ± 3.51*
BK	68.46 ± 1.02#	30.11 ± 2.82#
Inhibitor	66.95 ± 1.81#	31.05 ± 3.84#

Compared with normal group, *P < 0.05; compared with model group, #P < 0.05.

### 3.3. Cell apoptosis

**Figure 3:**
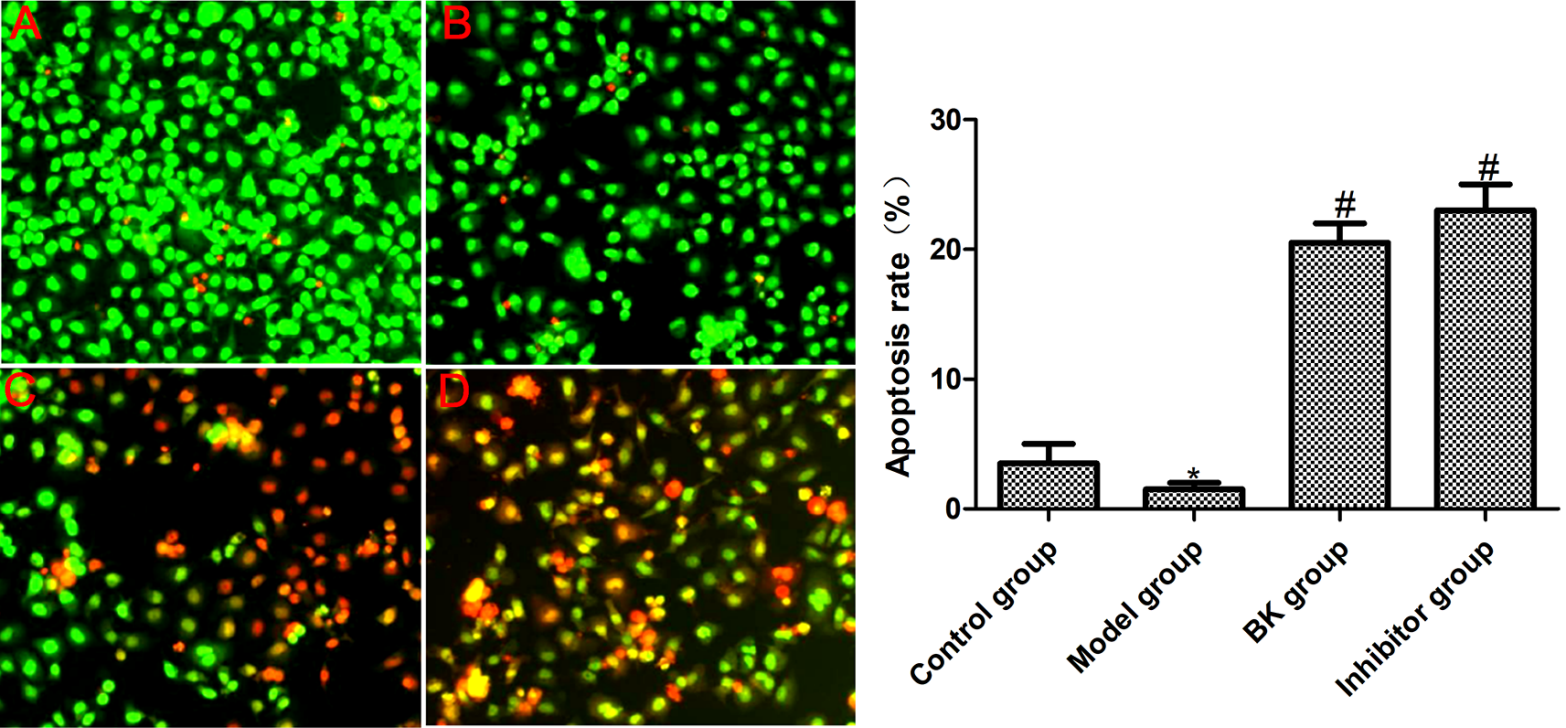
Cell apoptosis detected by AO/EB dual staining ( x ± s , n = 5). A: Control group; B: model group; C: BK group; D: inhibitor group. Compared with normal group, *P < 0.05; compared with model group, #P < 0.05.

AO can penetrate cells with intact cell membranes, enter the nucleus, and bind DNAs on chromosomes to emit bright green fluorescence. In contrast, EB can only pass through cells with damaged cell membranes, enter the nucleus, and bind DNAs on chromosomes to emit orange fluorescence (Metz et al., 2019). AO/EB dual staining (Figure 3) exhibited that a small number of cells in the normal group showed orange fluorescence, while the model group exhibited green fluorescence in the nucleus without apoptosis. In addition, BK and inhibitor groups displayed obvious lumpy orange fluorescence, indicating that there was a large number of apoptotic cells.

### 3.4. Col-1 expressions in cells

**Figure 4:**
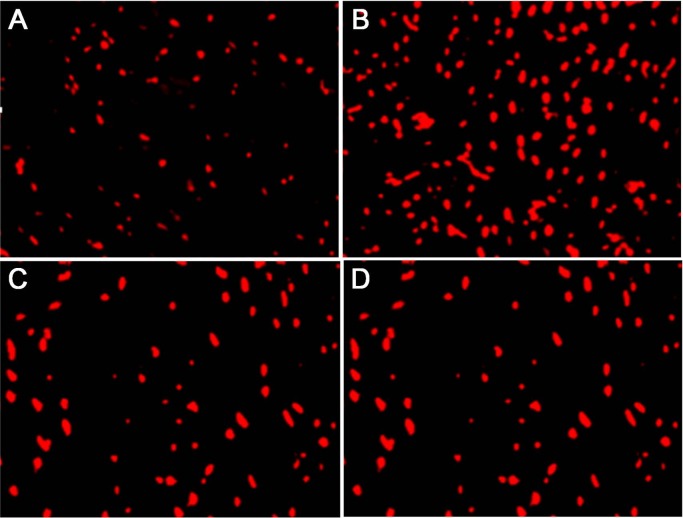
Col-1 expressions in cells detected by immunofluorescence assay ( x ± s , n = 5). A: Control group; B: model group; C: BK group; D: inhibitor group.

**Figure 5:**
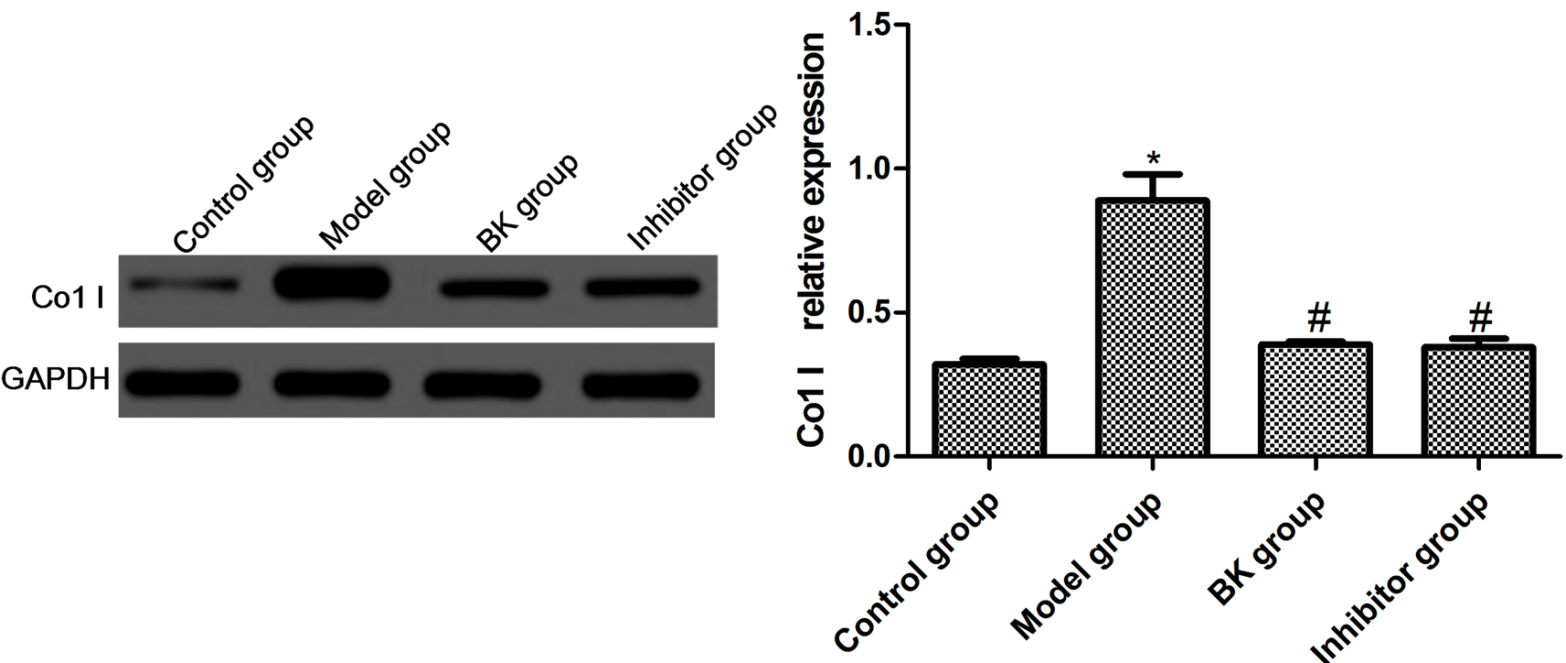
Col-1 expressions in cells detected by Western blot ( x ± s , n = 5). Compared with normal group, *P < 0.05; compared with model group, #P < 0.05.

Col-1 is the marker protein of renal cell fibrosis. In this study, the changes in Col-1 expression were detected by immunofluorescence assay. Compared with the normal group, the expression of Col-1 in the model group significantly increased after stimulation by TGF-β1 (P = 0.02). Compared with the model group, the expressions in BK and inhibitor groups declined significantly (P = 0.01), but the latter two groups had similar expressions (P = 0.09) (Figures 4 and 5).

### 3.5. TGF-β1, p-Smad2, and p-Smad7 protein expressions in cells

**Figure 6:**
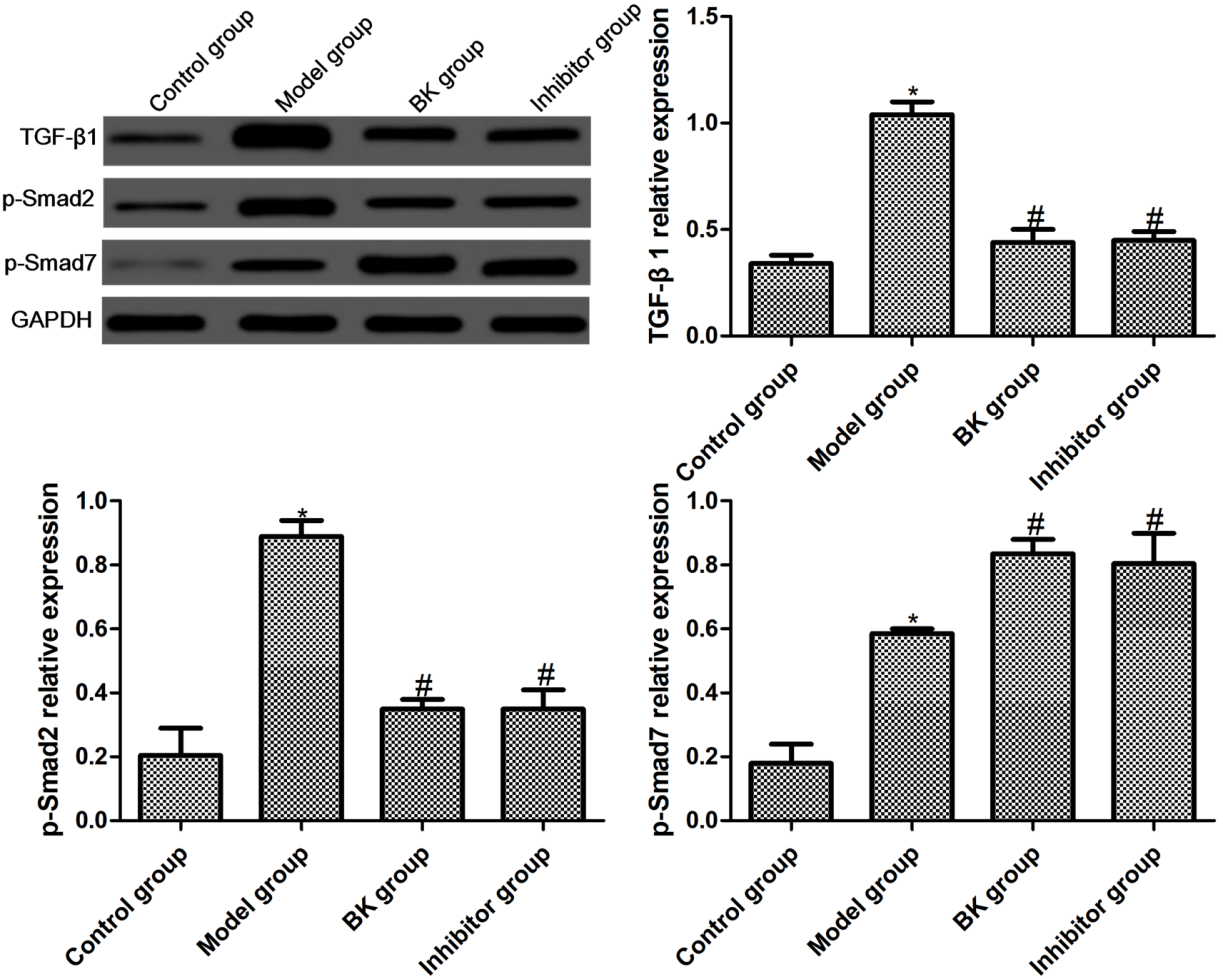
TGF-β1, p-Smad2, and p-Smad7 protein expressions in cells detected by Western blot ( x ± s , n = 5). Compared with normal group, *P < 0.05; compared with model group, #P < 0.05.

The protein expressions of TGF-β1, p-Smad2, and p-Smad7 in each group were detected by Western blotting. Compared with the normal group, the protein expressions of TGF-β1, p-Smad2, and p-Smad7 were significantly elevated in the model group (P = 0.01, P = 0.02, P = 0.00). The protein expressions of TGF-β1 and p-Smad2 in BK and inhibitor groups dropped significantly (P = 0.01, P = 0.00), while the protein expression of p-Smad7 rose significantly (P = 0.02). There were no significant differences between BK group and inhibitor group (P = 0.07) (Figure 6).

## 4. Discussion

Diabetic nephropathy (DN) is one of the common chronic complications of diabetes mellitus, the main cause of end-stage renal failure, and one of the main causes of death and disability. Thus, it has seriously threatened the physical and mental health and quality of life of diabetic patients (Zhu et al., 2018). DN is typified by the thickening of the glomerular basement membrane, the proliferation of mesangial cells, the accumulation of extracellular matrix to block the glomerular capillary lumen, and the loss of glomerular filtration function (Wu et al., 2018). Hyperglycemia plays a fundamental role in the onset and progression of DN, so good controlling of blood glucose level can reverse or relieve early diabetic kidney diseases. Additionally, reversing the proliferation state of glomerular mesangial cells in a high glucose environment and the high secretion of extracellular matrix is a critical factor for the treatment of DN. The most important active kinin is BK, which works mainly in the form of local hormones through two different types of receptors, i.e. B1 receptor and B2 receptor (autocrine and paracrine pathways). B1 receptor is rarely expressed in normal tissues or healthy animals. Instead, it is mainly expressed during stimulation and inflammation by bacterial lipopolysaccharides and interleukins, which may be related to inflammatory response. B2 receptor, which exists in normal organisms with a high density, is sensitive to BK. It is generally accepted that B2 receptor mediates most cardiovascular effects of kinin, electrolyte metabolism, and organ protection (Koçer et al., 2018; Nokkari et al., 2018). Renal interstitial fibrosis (RIF) is the common pathological basis for the development of many progressive kidney diseases into organ failure. However, the pathogenic mechanism has not been fully clarified. Excessive accumulation of collagen fibers caused by the abnormal proliferation of glomerular mesangial cells is the main pathological change of RIF (Hsieh et al., 2018). Therefore, finding effective means to suppress the abnormal proliferation of glomerular mesangial cells is of great clinical significance. 

As a small glycoprotein, BK is an effector peptide of the kallikrein-kinin system, which is widely distributed in the kidney, salivary gland, pancreas, sweat gland, central nervous system, and other tissues. It is mainly used for the clinical treatment of acute ischemia-reperfusion myocardial infarction (Zhu et al., 2019). Besides, BK can also protect various organs in the process of fibrosis (Acuña et al., 2018). Cui et al. (2017) reported that BK played a notable protective role in the organ fibrosis of aging rats. Fahmy et al. (2019) found that increasing BK intake in a short term evidently alleviated liver fibrosis in rats with bile congestion. Moreover, Deres et al. (2019) found that BK obviously protected spontaneously hypertensive rats from myocardial and vascular fibrosis. However, the antifibrotic role of BK has never been applied to the treatment of RIF hitherto. Herein, TGF-β1 stimulated the proliferation of rat glomerular mesangial cells, HBZY-1, whereas BK obviously inhibited cell proliferation, blocked the cell cycle in G1 phase, facilitated apoptosis, and reduced the expression of Col-1. The above results provide a new approach for the application of BK in the antifibrotic treatment of chronic renal diseases.

The TGF-β1/Smad signaling pathway has attracted widespread attention in studies on renal fibrosis. TGF-β1 is one of the most crucial factors for renal fibrosis. High expression of TGF-β1 is the central link of RIF formation, especially in hyperplastic glomerular mesangial cells (Cheng et al., 2019). Shi et al. (2018) verified that TGF-β1 stimulation was able to markedly facilitate the proliferation and fibrotic transformation of human glomerular mesangial cells in vitro. Smad proteins play a vital role in the formation of extracellular matrix in RIF. Smad2, a receptor protein of TGF-β1, can transfer signals to the nucleus to initiate the expression of collagen genes. Smad7 is an antagonist protein of serine/threonine kinase, which prevents the phosphorylation of activated Smad2. Kim et al. (2008) found that the TGF-β-Smad pathway was involved in the BK-stimulated α-SMA expression in human adipose tissue-derived mesenchymal stem cells. Wang et al. (2014) reported that inhibiting TGF-β1/Smad prevented human mesangial cells from proliferation triggered by platelet-derived growth factor-BB. Additionally, Ma et al. (2018) verified that repressing the TGF-β1/Smad signaling pathway relieved renal fibrosis in rats. Wei et al. (2018) reported that BK affected TGF‑β1‑induced epithelial‑mesenchymal transition by upregulating the expression of Smad7 and downregulating that of pSmad3 in the TGF‑β/Smad pathway, probably as an effective therapy for proliferative vitreoretinopathy. Furthermore, Zhang et al. (2019) demonstrated that suppressing the expression of Smad2 remarkably promoted high glucose-induced fibrosis of human renal tubular epithelial cells. 

In this study, after rat glomerular mesangial cells, HBZY-1, were stimulated by TGF-β1, the number of mitotic cells in the model group increased significantly, the proliferation rate rose remarkably, the apoptosis rate notably declined, and the expression of Col-1 was markedly raised. BK and inhibitor groups had significantly reduced cell proliferation rate and elevated apoptosis rate. Moreover, Western blotting showed that the protein expressions of TGF-β1 and p-Smad2 dramatically declined, but the protein expression of p-Smad7 obviously rose in BK group and inhibitor group. Therefore, BK may suppress the proliferation of rat glomerular mesangial cells and promote apoptosis by modulating the TGF-β1/Smad signaling pathway.

## 5. Conclusion

In summary, BK regulates the proliferation, apoptosis, and cycle of glomerular mesangial cells and other physiological processes by suppressing the TGF-β1/Smad signaling pathway. Further studies regarding whether BK modulates the proliferation and apoptosis of glomerular mesangial cells via pathways other than TGF-β1/Smad are ongoing in our group.
